# Prognostic role of Naples Prognostic Score in lung cancer: a meta-analysis

**DOI:** 10.1186/s13019-026-04011-1

**Published:** 2026-04-12

**Authors:** Shicheng Liu, Hongzhen Zhao, Zhaohui Han, Dahu Ren, Zengming Wang, Chunyan Zhao, Xiaopeng Zhang, Qingtao Zhao, Guochen Duan

**Affiliations:** 1 Department of Thoracic Surgery, Hebei General Hospital, Xinhua District, Hebei Province, NO. 348 Heping West Road, Shijiazhuang, 050057 People’s Republic of China; 2 Graduate School, Hebei Medical University, Shijiazhuang, 050017 People’s Republic of China

**Keywords:** Naples prognostic score, Lung cancer, Prognosis, Meta-analysis

## Abstract

**Purpose:**

The prognostic value of the Naples prognostic score in lung cancer remains controversial. Therefore, we performed a meta-analysis of relevant published studies to determine the prognostic value of the Naples prognostic score in patients with lung cancer.

**Methods:**

We conducted a systematic search of relevant studies in PubMed, Ovid, the Cochrane Library, and Web of Science databases. Data and characteristics of each study were extracted and hazard ratios (HRs) at 95% confidence intervals (95% CI) were calculated to estimate effects. A meta-regression analysis was used to assess the prognostic value of the Naples Prognostic Score in patients with lung cancer.

**Results:**

A total of 1691 patients from six studies were included in this meta-analysis, with a combined HR of 3.357 (95% CI: 1.964–5.738, P < 0.001); the results suggest that a high Naples Prognostic Score predicts a shorter overall survival (OS) for patients.

**Conclusion:**

This meta-analysis suggests that a high Naples Prognostic Score may be a predictor of poor prognosis in lung cancer patients. Further large cohort studies are needed to confirm these findings.

**Supplementary Information:**

The online version contains supplementary material available at 10.1186/s13019-026-04011-1.

## Introduction

Lung cancer is one of the most common malignancies and the leading cause of cancer-related deaths globally [1]. Relevant research shows that [2], in 2020, there were 2.2 million new lung cancer cases and 1.8 million deaths worldwide, accounting for about one in ten (11.4%) cancer diagnoses and one in five (18.0%) cancer-related deaths. In recent years, advances in our understanding of lung cancer risk, development, immune control, and treatment options have enabled lung cancer patients to increasingly receive precise, individualized diagnosis and treatment. However, the overall prognosis of lung cancer remains unsatisfactory due to local tumor recurrence and distant metastasis [3]. Therefore, it becomes critical to identify reliable and valid markers to better predict prognosis.

In recent years, the Naples Prognostic Score (NPS) has entered the public eye as a new inflammatory nutrition scoring system, calculated from the patient’s preoperative serum albumin (ALB), total cholesterol (TC), neutrophil-to-lymphocyte ratio (NLR) and lymphocyte-to-monocyte ratio (LMR), reflecting the patient’s systemic inflammatory response and nutritional status [4]. The NPS was identified as a prognostic indicator for various cancers, such as breast cancer, [5] esophageal cancer [6], gastric cancer [7], ampullary carcinoma [8], pancreatic cancer [9] and colorectal cancer [10]. The prognostic value of the NPS in lung cancer was also investigated. [11–16]. However, these studies reported inconsistent results due to differences in study design and sample size. Therefore, it is unclear whether the NPS is a valid prognostic indicator for lung cancer. In this study, we searched existing studies and performed a meta-analysis to assess the prognostic role of the Naples Prognostic Score in lung cancer.

## Materials and methods

### Study design and reporting standards

This systematic review and meta-analysis was conducted and reported in accordance with the Preferred Reporting Items for Systematic Reviews and Meta-Analyses (PRISMA) 2020 statement.

### Search strategy

We conducted a comprehensive literature search of the articles through the following databases, with no date limits: PubMed, Ovid, the Cochrane Library, and the Web of Science databases. The search was updated to November 2024. The main search terms included: (NPS or Naples prognostic score) and (lung cancer or lung carcinoma or NSCLC or SCLC). Relevant articles in the list of references were also examined.

### Data extraction

All candidate articles were evaluated and extracted by three independent researchers (Shicheng Liu, Hongzhen Zhao and Zhaohui Han). The articles that couldn’t be classified by title and abstract only were full-text reviewed by retrieval. If disagreement occurred, the three researchers will discuss and reach a consensus with the fourth investigators (Duan Guochen).

For each study, the following items were recorded: first author’s name, year of publication, country, sample size, gender, follow-ups, treatment strategy, cancer type and HRs with 95%CI. Given that all studies were retrospective studies, both researchers used the Newcastle–Ottawa Scale (NOS) to assess the quality of each included study. The NOS consists of three parts: selection (0–4 points), comparability (0–2 points), and outcome assessment (0–3 points). NOS score ≥ 6 is considered to be a high-quality study.

### Inclusion and exclusion criteria

Studies were included if they met all the following criteria: (1) The study population consisted of patients with pathologically confirmed lung cancer; (2) The NPS was calculated based on preoperative serum levels of ALB, TC, NLR and LMR; (3)The study reported the association between NPS and at least one of the following survival outcomes: overall survival (OS), progression-free survival (PFS), or disease-free survival (DFS). Sufficient data were provided to extract or calculate hazard ratios (HRs) with 95% confidence intervals (95% CI); (4) The article was a full-text original research paper published in English.

Studies were excluded for any of the following reasons: (1) Publication types such as abstracts, letters, editorials, reviews, meta-analyses, case reports, or non-clinical studies; (2) Studies with overlapping patient cohorts or duplicate publications (only the most comprehensive or recent study was retained); (3) Studies for which the full text was unavailable or that were published in languages other than English.

### Statistical analysis

We directly obtained HR and 95%CI from each literature or estimated these data from each study according to the method illustrated by Parmar et al. [17]. The association of the NPS score and the prognosis of lung cancer patients was evaluated by these data. A HR > 1 indicated a worse prognosis in lung cancer patients with high NPS. We conducted a heterogeneity test on included studies using the I^2^ test. The fixed effects model was applied to conduct the meta-analysis if no remarkable inter-study heterogeneity (P > 0.1 or I^2^ < 50.0%). If significant heterogeneity (P < 0.1 or I^2^ ≥ 50.0%) was found, the random effects model was applied for the meta-analysis. If there is considerable heterogeneity, meta-regression analysis will be performed using the restricted maximum likelihood (REML) method to explore the potential impact of the following continuous or categorical covariates: year of publication, sample size, median age, proportion of non-small cell lung cancer (NSCLC) patients, and proportion of advanced-stage (III/IV) patients. Subgroup analysis based on these and other clinical factors will also be conducted. Publication bias was estimated by Begg’s rank correlation test and Egger’s regression asymmetry test [18]. To assess the robustness of the results, a sensitivity analysis was conducted using R software (version 4.1.0) for statistical computation. P < 0.05 was considered statistically significant.

## Results

### Study characteristics

**Fig. 1:**
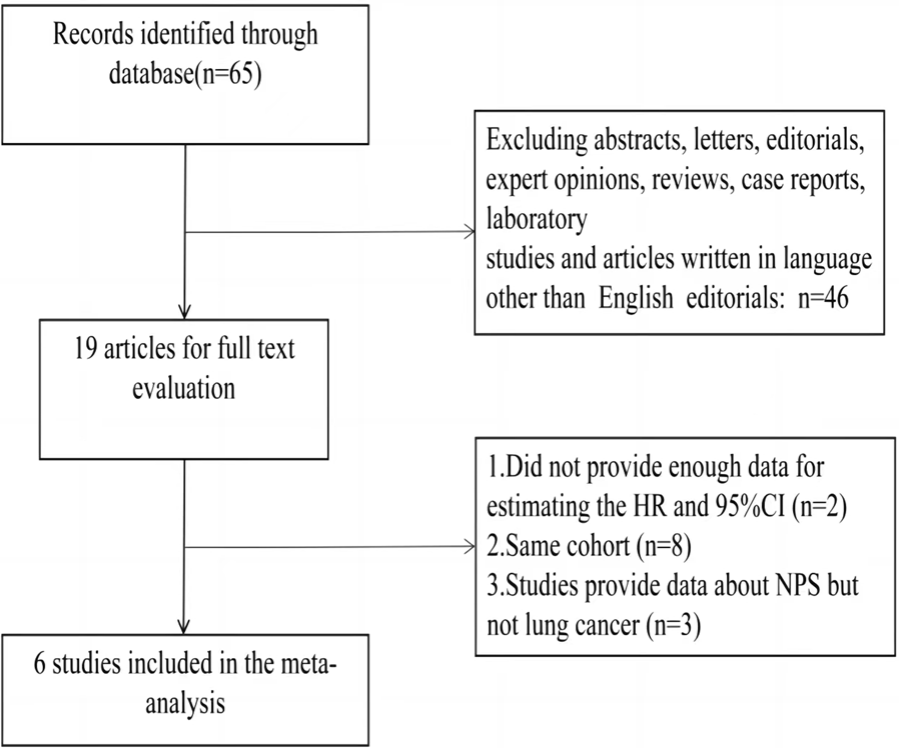
Flow chart of the included studies

The process of study selection has been described in the flow chart (Fig. 1). The initial search strategies retrieved a total of 65 articles. After a careful examination of these articles, 6 studies with a total of 1,691 patients published between 2021 and 2022 were eventually enrolled in our meta-analysis. In 6 articles, the correlation between NPS and OS was studied. Among them, 3 articles studied the correlation between NPS and PFS; another 3 studies the correlation between NPS and DFS. HRs and 95% CI were reported directly in all the studies. 2 of these studies enrolled ≤ 200 patients and 4 studies had > 200 patients. All studies were retrospective cohort studies. All of the studies were obtained from China. The characteristics of the included studies were shown in Table 1.

**Table 1:** Main characteristics of all the studies included in the meta-analysis

Author	Year	Region	No (M/F)	Follow-up (months) (median and range)	Treatment	Age (years) (median and range)	NPS	Outcome	Stage	Type	HR	NOS
Ren et al [11]	2022	China	319(162/157)	32	Surgery Chemotherapy radiotherapy TKI treatment	61 (interquartile range, IQR: 13)	0, 1, 2	OS/DFS	I/II/III	NSCLC	R (U/M)	6
Li et al [12]	2021	China	457(283/174)	50 (12–66)	Surgery	63 (IQR = 59–69)	0, 1, 2	OS/DFS	I/II	NSCLC	R (U/M)	7
Chen et al [13]	2022	China	128(97/37)	12.3	Surgery Chemotherapy radiotherapy	65 (14–82)	0, 1, 2	OS/PFS	Limited stage Extensive stage	SCLC	R (U/M)	6
Guo et al [14]	2021	China	206(120/86)	37 (13–59)	Chemotherapy radiotherapy	62 (36–84)	0, 1, 2	OS/PFS	III	NSCLC	R (U/M)	6
Peng et al [15]	2022	China	395(252/143)	32 (1–60)	Surgery Chemotherapy radiotherapy TKI treatment	(24–86)	0, 1, 2	OS/PFS	I/II/III/IV	NSCLC	R (U/M)	7
Xuan et al [16]	2022	China	186(85/101)	32 (6–55)	Radiotherapy	57 (39–72)	0, 1, 2	OS/DFS	IV	NSCLC	R (U/M)	6

M, male; F, female; NPS: Naples Prognostic Score; HR, hazard ratio; NOS, Newcastle–Ottawa Scale; OS, overall survival; DFS, disease-free survival; NSCLC, non-small-cell lung cancer; R, reporting; U, univariate analysis; M, multivariate; PFS, progression-free survival; SCLC, non-small-cell lung cancer; TKI, tyrosine kinase inhibitor

### The relationship between NPS and OS of lung cancer

**Fig. 2:**
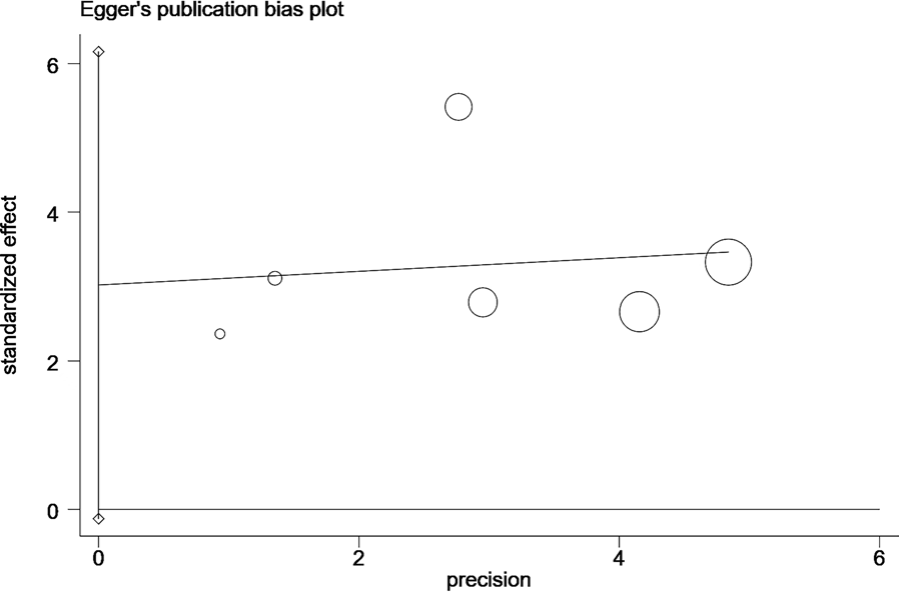
Meta-analysis of the association between NPS and OS of lung cancer. Weights are from random-effects analysis

All 6 studies reported a correlation between NPS and OS in lung cancer patients. Because of significant heterogeneity (I^2^ = 69.8%, Ph = 0.005), Therefore, a random effects model was applied. The results show that high NPS predict worse OS outcomes (HR: 3.357, 95% CI: 1.964–5.738, P < 0.001). (Fig. 2).

### The relationship between NPS and PFS and DFS in lung cancer

**Fig. 3:**
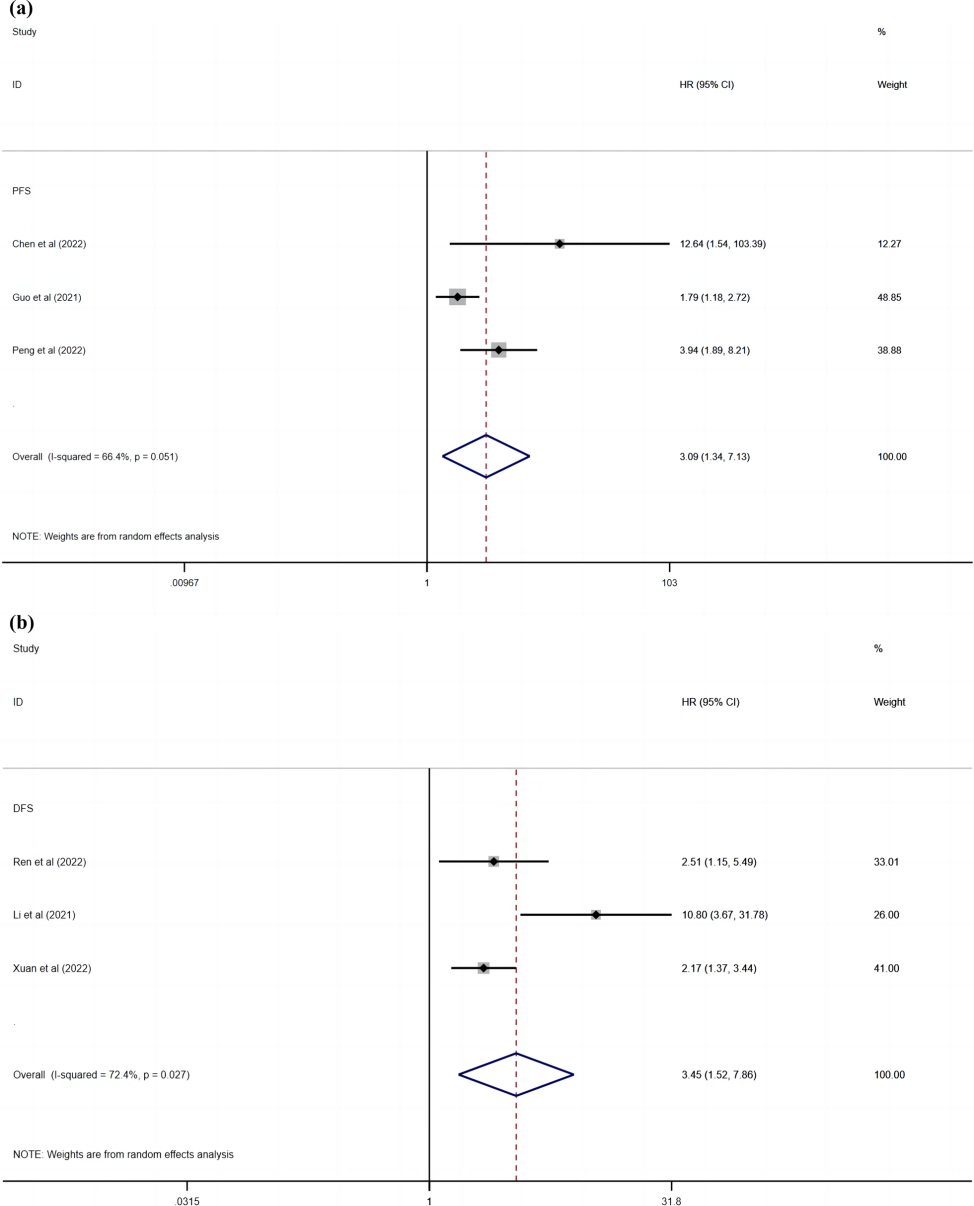
(**a**) Meta-analysis of the association between NPS and PFS of lung cancer. (**b**) Meta-analysis of the association between NPS and DFS of lung cancer. Weights are from random-effects analysis.

Three studies reported the relationship between NPS and PFS in lung cancer patients, and our meta-analysis showed that patients with high NPS were associated with shorter PFS (random effects model obtained by HR: 3.094, 95% CI: 1.344–7.126, P = 0.008; Fig. 3a), with high heterogeneity (I^2^ = 66.4%, Ph = 0.051). Three other studies assessed the relationship between NPS and DFS, and our results show that high NPS predict worse DFS outcomes (obtained by the random effects model HR: 3.455, 95% CI: 1.518–7.862, P = 0.003; Fig. 3b), with substantial heterogeneity (I^2 ^= 72.4%, Ph = 0.027).

### Subgroup analysis

We further explored potential causes of heterogeneity. For OS, subgroup analyses were performed by mode of treatment (treatment with surgery and treatment without surgery), type (non-small cell lung cancer [NSCLC] and small cell lung cancer [SCLC]), sample size (> 200 and ≤ 200), stage (advanced stage: III/IV and stages I to IV: I/II/III/IV). Most subgroup analyses did not significantly alter the prognostic role of NPS scores in OS (Table 2). However, for subgroup analyses with a sample size ≤ 200, patients with high NPS scores were not significantly associated with shorter OS, with a combined HR of 3.671(Random-effects model, 95% CI: 0.625–21.558, P = 0.150).

**Table 2:** Subgroup analysis of the association between the Naples Prognostic Score (NPS) and overall survival (OS) in lung cancer patients

Analysis	N	References	Random-effects model		Fixed-effects model		Heterogeneity	
			**HR(95%CI)**	***P***	**HR(95%CI)**	***P***	***I***^**2**^	***Ph***
OS	6	11, 12, 13, 14, 15, 16	3.357 (1.964–5.738)	0.000	2.586 (2.007–3.332)	0.000	69.8%	0.005
Subgroup 1:Treatment methods including surgery	4	11, 12, 13, 15	5.494 (2.596–11.626)	0.000	4.728 (3.018–7.406)	0.000	52.7%	0.096
reatment methods not including surgery	2	14, 16			1.949 (1.433–2.650)	0.000	0.0%	0.883
Subgroup 2:sample size > 200	4	11, 12, 14, 15	3.760 (1.843–7.671)	0.000	2.845 (2.099–3.855)	0.000	75.7%	0.006
sample size ≤ 200	2	13, 16	3.671 (0.625–21.558)	0.150	2.077 (1.311–3.292)	0.002	66.4%	0.085
Subgroup 3: stage: I/II/III/IV		11, 12, 13, 15	5.494 (2.596–11.626)	0.000	4.728 (3.018–7.406)	0.000	52.7%	0.096
stage: III/IV		14, 16			2.065 (1.523–2.800)	0.000	0.0%	0.781
Subgroup 4:								
NSCLC	5	11, 12, 14, 15, 16	3.097 (1.816–5.282)	0.000	2.526 (1.957–3.261)	0.000	72.1%	0.006
DFS	3	11, 12, 16	3.455 (1.518–7.862)	0.003	2.716 (1.871–3.945)	0.000	72.4%	0.027
PFS	3	13, 14, 15	3.094 (1.344–7.126)	0.008	2.286 (1.599–3.267)	0.000	66.4%	0.051

N, number; HR, hazard ratio; CI, confidence interval; OS, overall survival; NSCLC, non-small-cell lung cancer; DFS, disease-free survival; PFS, progression-free survival

Pooled HRs and 95% CI were calculated using a fixed-effects model unless otherwise specified. The random-effects model was applied for subgroups where significant heterogeneity was present (I^2^ ≥ 50% or Ph < 0.1)

### Sensitivity analysis

**Fig. 4:**
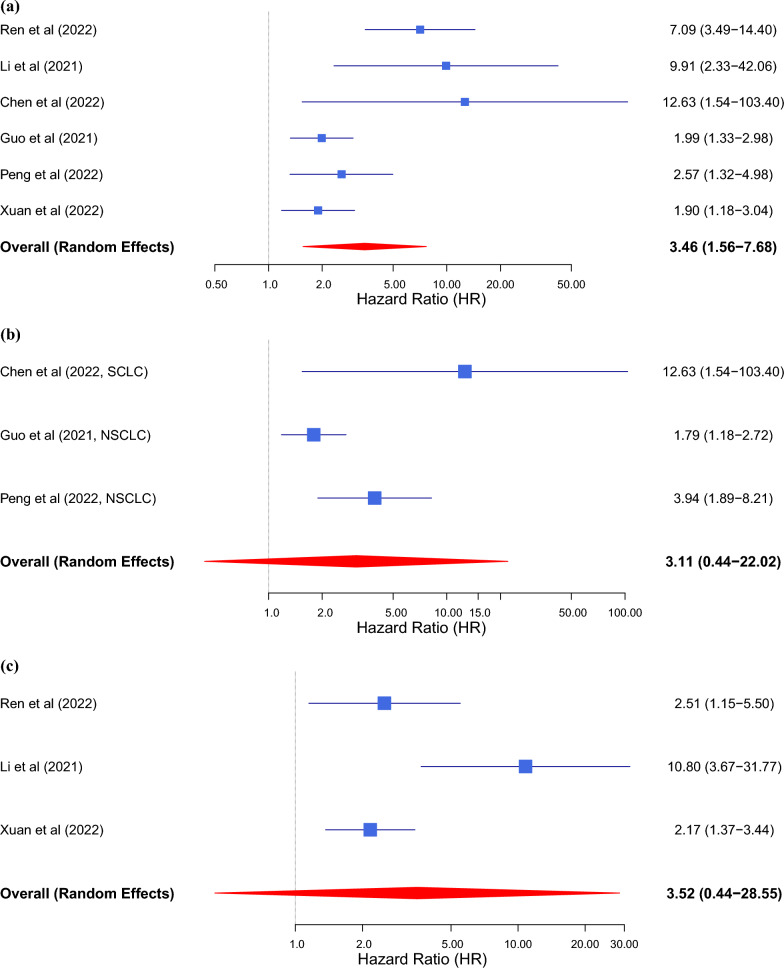
(**a**) Sensitivity analysis of OS in this meta-analysis. (**b**) Sensitivity analysis of PFS in this meta-analysis. (**c**) Sensitivity analysis of DFS in this meta-analysis

Sensitivity analysis was performed using the leave-one-out method by sequentially removing each study and recalculating the pooled results for OS(Fig. 4a), PFS(Fig. 4b), and DFS(Fig. 4c). The results did not substantially change, showing the reliability and stability of our results. Meanwhile, subgroup analysis was also conducted to explore the potential factors that are responsible for heterogeneity in OS. The results showed that the above factors could partly explain the heterogeneity but did not reach statistical significance Table 2**.**

### Publication bias

**Fig. 5:**
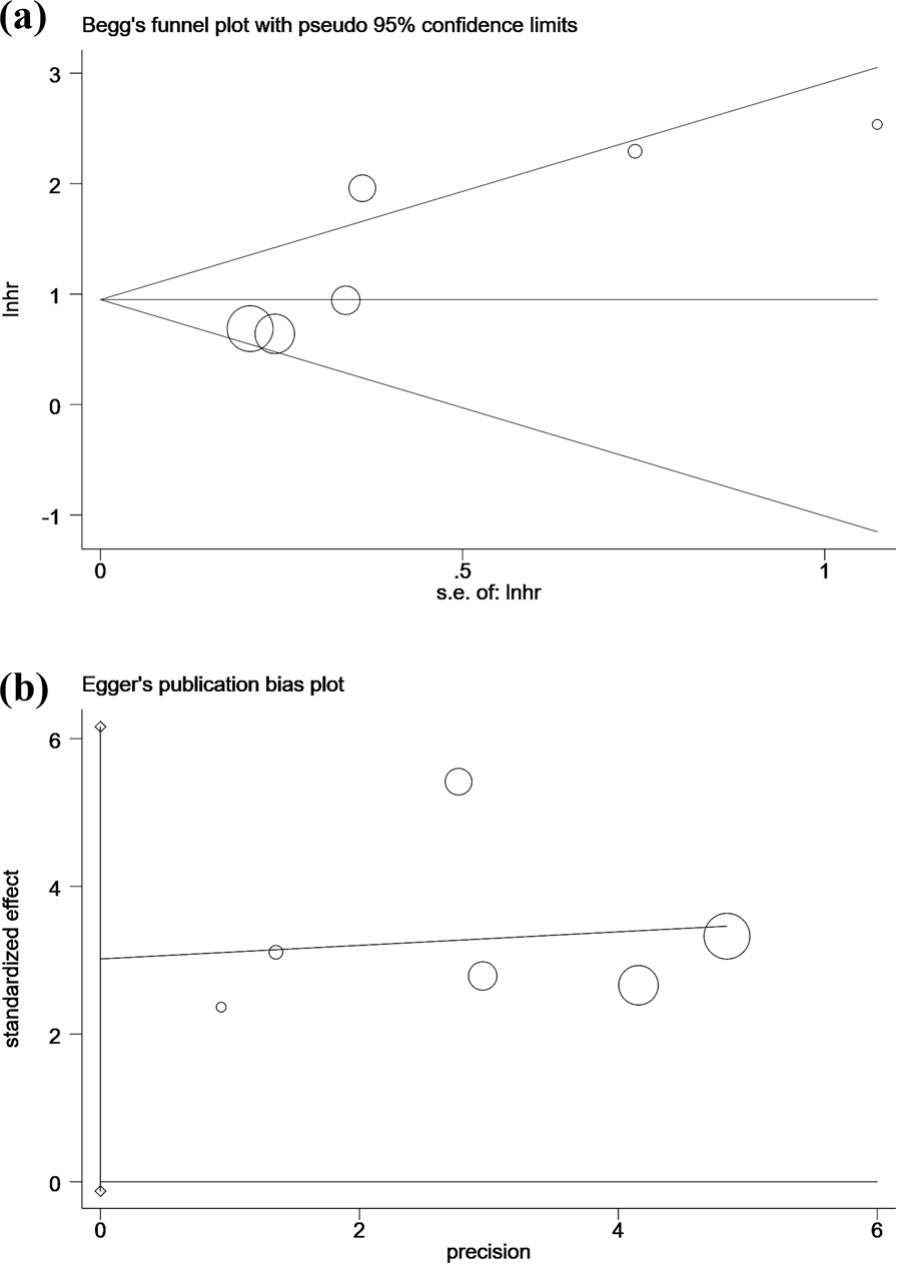
Begg’s funnel plot and Egger’s funnel plot in the meta-analysis of lung cancer. (**a**) OS for NPS in Begg’s funnel plot in the meta-analysis of lung cancer. (**b**) OS for NPS in Egger’s funnel plot in the meta-analysis of lung cancer

In order to estimate the publication bias, the Begg’s funnel plot and Egger’s linear regression test were applied. No publication bias was detected for OS in Begg’s test (Pr >|z|= 0.133; Fig. 5a) and Egger’s test (P >|t |= 0.056; Fig. 5b).

## Discussion

Our meta-analysis, pooling data from 1,691 lung cancer patients across 6 studies, demonstrated that a high Naples prognostic score (NPS) was significantly associated with poorer OS in lung cancer patients (HR: 3.357, 95%CI: 1.964–5.738, P < 0.001, Fig. 2).

We acknowledge the presence of considerable statistical heterogeneity in our analyses (I^2^ ranging from 66 to 72%). Although we conducted subgroup analyses and meta-regression exploring covariates such as sample size, treatment modality, cancer type, and disease stage, no single covariate was conclusively identified as the dominant source of this heterogeneity. This residual heterogeneity suggests that unmeasured factors, such as differences in patient comorbidities, specific treatment regimens, or NPS assessment timing, may influence the prognostic effect size. Therefore, the pooled HR estimates should be interpreted with caution, recognizing a degree of uncertainty in their precision.

Nevertheless, it is noteworthy that the direction of effect was consistent across all included studies, with high NPS consistently linked to worse survival outcomes. Both subgroup and sensitivity analyses did not substantially alter the overall conclusion. Thus, despite the statistical heterogeneity, the current evidence robustly supports a significant and positive association between elevated NPS and adverse prognosis in lung cancer patients. Further investigation is needed to clarify the modifiers of this relationship.

There is ample evidence that [19–23]. Many assessment tools based on conventional systemic inflammation and nutritional biomarkers have reliable clinical significance in various types of cancer [24–26], Although their importance in cancer etiology has been demonstrated, the exact mechanism between them and tumors has not been determined.

As a new type of risk scoring system, NPS includes four parts: serum albumin (ALB), total cholesterol (TC), neutrophil-to-lymphocyte ratio (NLR) and lymphocyte-to-monocyte ratio (LMR), which fully considers the patient’s inflammatory response, immune capacity and nutritional status, and its prognostic effect on lung cancer has also been revealed in the past two years. Neutrophils, as a widely existing inflammatory cell in the human body, can secrete related cytokines and chemokines during the occurrence and development of cancer, promote the proliferation and metastasis of tumor cells, and also promote the formation of new blood vessels in tumor tissue. And can increase the infiltration degree of blood vessels in tumor tissue, can also inhibit the immune activity of T lymphocytes and natural killer cells (natural killer cell, NK), forming an immunosuppressive environment conducive to the survival of tumor cells, thereby promoting the development of malignant tumor. [27–29]. Monocytes can differentiate into tumor-associated macrophages during cancer development, stimulate tumor angiogenesis by secreting oncostatin-M and vascular endothelial growth factor (VEGF), enhance the fluidity and invasiveness of tumor cells, thereby accelerating tumor cell progression and dissemination [30, 31]. Different from the former two, lymphocytes, as an important part of the human immune system, kill cancer cells through tumor immune monitoring and cytotoxic activity in the tumor microenvironment, so as to inhibit the spread and migration of tumor cells [32, 33]. NLR and LMR objectively reflect the inflammatory and immune status of the host. Increased NLR and decreased LMR are usually accompanied by an increase in neutrophils or a significant decrease in peripheral blood lymphocytes. Elevated NLR and decreased LMR are often associated with higher mortality and poorer prognosis in various malignancies [34, 35]. Nutrition is closely related to tumor growth and progression, and malnutrition is often one of the reasons for poor prognosis. ALB is a marker for assessing nutritional status, and lower serum albumin concentrations have been shown to predict an adverse outcome in many nutrition scoring systems [36, 37]. Cholesterol is an important part of cell membranes, and low cholesterol levels reduce the fluidity of cell membranes, while also impairing the ability of cell surface receptors to transmit transmembrane signals, so its reduction may indicate a poor prognosis in patients [38]. Therefore, the NPS fully reflects the body’s inflammatory response, immune capacity, and nutritional status, and is an effective prognostic marker that can help optimize clinical decision-making for lung cancer treatment. However, there are only a few studies evaluating the prognostic value of the NPS in lung cancer patients, and systematic analysis is lacking. Therefore, meta-analysis is the most effective method to gain insight into the prognostic impact of NPS on lung cancer patients.

The consistent association between high NPS and poor survival across studies underscores its potential utility as a preoperative risk stratification tool. Identifying patients with elevated NPS could flag a subgroup at higher risk for adverse outcomes, prompting more vigilant monitoring.From a mechanistic perspective, our findings generate hypotheses for future interventional research. The components of NPS reflect inflammation, immunity, and nutrition—all modifiable factors. It is plausible to hypothesize that for patients identified as high-risk by NPS, a multimodal prehabilitation program addressing these domains (e.g., anti-inflammatory strategies, immunonutrition, and physical conditioning) might improve surgical resilience and outcomes. Similarly, one could speculate that such high-risk patients might derive greater benefit from less invasive surgical approaches (e.g., video-assisted thoracoscopic surgery) to reduce physiological stress, though this remains to be tested prospectively.However, it is crucial to emphasize that our meta-analysis, synthesizing retrospective observational data, cannot directly support specific perioperative management changes. The causal relationship between modifying NPS components and improving survival is unproven. Therefore, the primary immediate clinical value of NPS lies in prognostic stratification and enriching patient counseling. The hypotheses generated herein—regarding targeted prehabilitation, surgical approach selection, and postoperative nutritional support—represent critical avenues for future prospective, interventional studies designed to determine whether NPS can effectively guide therapy and improve patient outcomes.

However, this meta-analysis have several limitations. First, the studies included in this study were retrospective in nature, which could easily lead to some biases such as information bias, misclassification bias, and selection bias. Second, the generalizability of our findings is inherently limited because all six studies were conducted exclusively in Chinese populations. While this provides consistent evidence within this context, it raises important questions about external validity. The prognostic utility of the NPS may vary across different ethnicities due to genetic predispositions, lifestyle factors, and variations in the prevalence of conditions affecting inflammatory or nutritional biomarkers. Furthermore, treatment strategies, perioperative care standards, and overall healthcare systems differ globally, all of which could influence the relationship between NPS and survival outcomes. Therefore, our pooled estimates primarily reflect the prognostic value of NPS in the Chinese healthcare setting and should be extrapolated to other populations with caution. Third, the modest number of studies and the presence of significant statistical heterogeneity. To address these limitations, future research should prioritize large-scale, prospective, and multicenter studies that include diverse ethnic populations from different geographic regions. Such studies are essential to validate the universal applicability of the NPS and to establish potential ethnicity- or region-specific cut-off values.Fourth, although we rigorously followed the PRISMA reporting guidelines, our study protocol was not prospectively registered in an international database (e.g. PROSPERO). This could introduce potential reporting bias.

## Conclusion

In conclusion, this meta-analysis shows that high NPS are significantly associated with poorer prognosis in lung cancer patients. High NPS was significantly associated with inferior OS (HR = 3.357, 95%CI:1.964–5.738, P < 0.001). Similarly, elevated NPS was correlated with shorter PFS (HR = 3.094, 95% CI:1.344–7.126, P = 0.008) and poorer DFS (HR = 3.455, 95% CI:1.518–7.862, P = 0.003).The NPS appears to be a convenient, reproducible, widely available and reliable method for predicting the survival status of lung cancer patients. In the future, large-scale, more well-designed prospective studies are needed to further validate the conclusion of the association between NPS and lung cancer prognosis.

## Supplementary Information

Supplementary Material 1

## Data Availability

No datasets were generated or analysed during the current study.
